# Bis[1-(2-hydroxy­ethyl)-2-methyl-5-nitro-1*H*-imidazole-κ*N* ^3^]silver(I) nitrate

**DOI:** 10.1107/S1600536808009860

**Published:** 2008-04-16

**Authors:** Hoong-Kun Fun, Samuel Robinson Jebas, T. Balasubramanian

**Affiliations:** a X-ray Crystallography Unit, School of Physics, Universiti Sains Malaysia, 11800 USM, Penang, Malaysia; b Department of Physics, National Institute of Technology, Tiruchirappalli 620 015, India

## Abstract

In the title compound, [Ag(C_6_H_9_N_3_O_3_)_2_]NO_3_, the Ag atom is bicoordinated in a distorted linear configuration by two 1-(2-hydroxy­ethyl)-2-methyl-5-nitro­imidazole ligands through one of the N atoms. The dihedral angle between the two imidazole rings is 16.1 (2)°. The O atoms of the nitrate anion are disordered over two positions; the site occupancy factors are 0.8 and 0.2. The ions are ­connected by C—H⋯O inter­actions, while two weak intra­molecular C—H⋯O inter­actions producing an *S*(6) ring motif are observed. The nitrate anion is linked to the hydroxyl groups of two neighbouring cations by O—H⋯O hydrogen bonds. The ions are packed into infinite chains along the [100] direction.

## Related literature

For related literature regarding pharmaceutical uses of nitro­imidazole derivatives, see: Credito *et al.* (2000 ▶); Edwards (1981 ▶); Mendz & Megraud (2002 ▶). For comparable crystal structures, see: Blaton *et al.* (1979 ▶); Gao *et al.* (2004 ▶); Ni *et al.* (2003 ▶); Pi *et al.* (2005 ▶); Tong & Chen (2000 ▶); Yang *et al.* (2005 ▶); You & Zhu (2004 ▶).
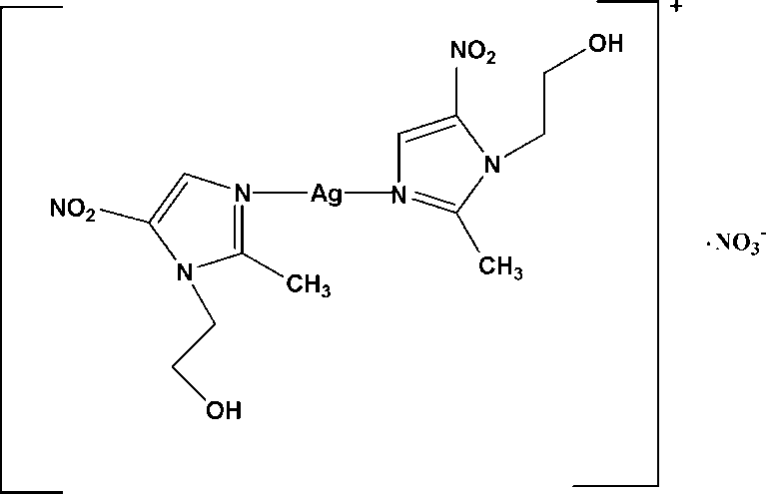


## Experimental

### 

#### Crystal data

[Ag(C_6_H_9_N_3_O_3_)_2_]NO_3_
*M* *_r_* = 512.2
Triclinic, 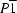

*a* = 6.6912 (1) Å
*b* = 11.6846 (3) Å
*c* = 12.9052 (3) Å
α = 63.707 (1)°
β = 88.820 (1)°
γ = 87.486 (1)°
*V* = 903.72 (3) Å^3^
*Z* = 2
Mo *K*α radiation
μ = 1.18 mm^−1^
*T* = 100.0 (1) K
0.74 × 0.22 × 0.1 mm

#### Data collection

Bruker SMART APEXII CCD area-detector diffractometer
Absorption correction: multi-scan (*SADABS*; Bruker, 2005 ▶) *T*_min_ = 0.476, *T*_max_ = 0.892
20384 measured reflections
6509 independent reflections
5958 reflections with *I* > 2σ(*I*)
*R*_int_ = 0.026

#### Refinement

*R*[*F* ^2^ > 2σ(*F* ^2^)] = 0.024
*wR*(*F* ^2^) = 0.059
*S* = 1.08
6509 reflections
291 parameters
H-atom parameters constrained
Δρ_max_ = 0.58 e Å^−3^
Δρ_min_ = −0.65 e Å^−3^

### 

Data collection: *APEX2* (Bruker, 2005 ▶); cell refinement: *APEX2*; data reduction: *SAINT* (Bruker, 2005 ▶); program(s) used to solve structure: *SHELXTL* (Sheldrick, 2008 ▶); program(s) used to refine structure: *SHELXTL*; molecular graphics: *SHELXTL*; software used to prepare material for publication: *SHELXTL* and *PLATON* (Spek, 2003 ▶).

## Supplementary Material

Crystal structure: contains datablocks global, I. DOI: 10.1107/S1600536808009860/ez2123sup1.cif

Structure factors: contains datablocks I. DOI: 10.1107/S1600536808009860/ez2123Isup2.hkl

Additional supplementary materials:  crystallographic information; 3D view; checkCIF report

## Figures and Tables

**Table 1:** Hydrogen-bond geometry (Å, °)

*D*—H⋯*A*	*D*—H	H⋯*A*	*D*⋯*A*	*D*—H⋯*A*
O1—H101⋯O9*B*^i^	0.75	2.01	2.685 (5)	150
O1—H101⋯O8*A*^i^	0.75	2.15	2.8872 (17)	164
O4—H1*O*4⋯O7*A*^ii^	0.75	2.02	2.7225 (18)	158
O4—H1*O*4⋯O8*B*^ii^	0.75	2.29	2.985 (5)	156
C3—H3*A*⋯O8*A*	0.93	2.27	3.0820	145
C4—H4*A*⋯O9*A*	0.93	2.42	3.0269	122
C8—H8*B*⋯O2	0.97	2.35	2.891 (2)	115
C10—H10*B*⋯O5	0.97	2.36	2.888 (2)	113

Symmetry codes: (i) 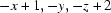; (ii) 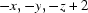.

**Fig. 1.**
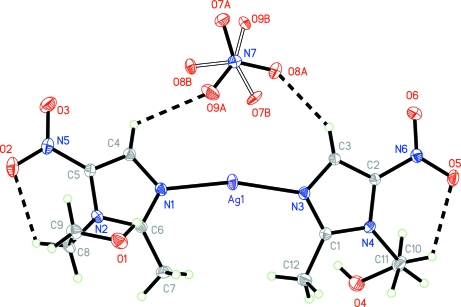
The asymmetric unit of the title compound, showing 50% probability displacement ellipsoids and the atomic numbering. Hydrogen bonds are shown as dashed lines.

**Fig. 2.**
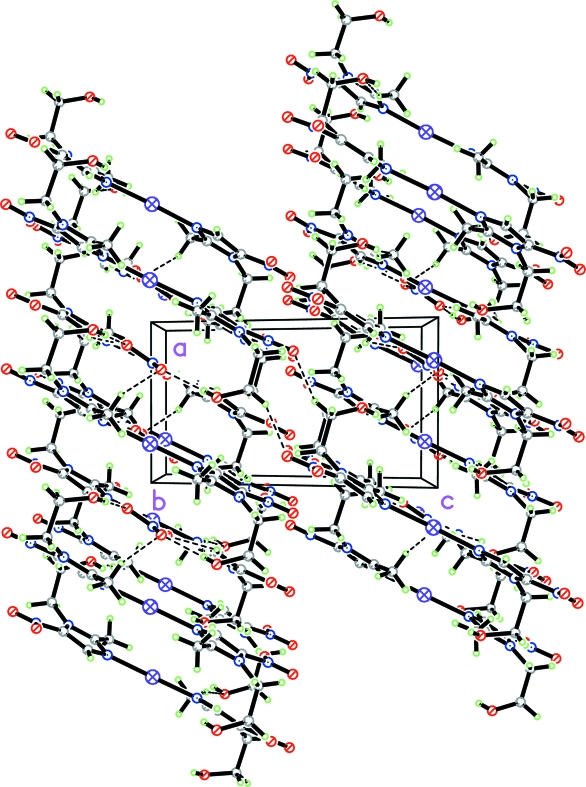
The crystal packing of the title compound, viewed down the b axis. Hydrogen bonds are shown as dashed lines.

## supplementary crystallographic information

### Comment

Nitroimidazole derivatives are of special interest due to their chemical and pharmacological properties. Nitroimidazoles are generally known as antiprotozoic and radiosensitizing drugs (Edwards, 1981). (1-(2-hydroxyethyl)-2-methyl-5-nitroimidazole), known as metronidazole in pharmaceuticals, is a widely used antibacterial drug (Credito *et al.*, 2000; Mendz & Megraud, 2002). The structure of the title compound (I) has been determined to examine the influence of the coordination of silver on the geometry of the heterocycle.

The structure consists of two 1-(2-hydroxyethyl)2-methyl-5-nitroimidazole ligands coordinating to the silver through the N atoms in a distorted linear configuration, indicated by the N3—Ag1—N1 angle of 165.34 (4) °. The bond lengths of Ag1—N3 = 2.147 (11) Å and Ag1—N1 = 2.148 (11) Å, are comparable to the values reported for similar silver coordinated complexes (Tong & Chen, 2000; Ni *et al.*, 2003; You & Zhu, 2004; Gao *et al.*, 2004). The bond lengths of the heterocyclic five membered rings are comparable with the values found in 1-(2-hydroxyethyl)-2-methyl-5-nitroimidazole in its un-coordinated form (Blaton *et al.*, 1979), iodometronidazole (Yang *et al.*, 2005), and chlorometronidazole (Pi *et al.*, 2005).

Both the hydroxyl oxygen atoms, O1 and O4, attached to the imidazole rings are twisted from the mean plane, with O1–C9–C8–N2 and O4–C11–C10–N4 torsion angles being -66.96 (14)° and -71.9 (14)° respectively. The nitro groups attached to the imidazole rings are slightly twisted, with O3—N5—C5—C4 and O6—N6—C2—C3 torsion angles of -10.8 (2)° and -13.3 (2)° respectively. Both imidazole rings are essentially planar, with the maximum deviation from planarity being 0.007 (1) Å for atom C1 and 0.003 (2) Å for atom N2. The nitrate anion is disordered over two positions with site occupancies of 0.8:0.2. The molecules in the asymmetric unit are interconnected by C—H···O hydrogen bonds. Intramolecular C—H···O hydrogen bonding results in an S(6) a ring motif, while two oxygen atoms of the major component of the disordered nitrate anion form C—H···O interations with the cation, forming an *R*^2^_2_(10) motif (Fig. 1). The nitrate ions are linked to the hydroxyl groups on neighbouring cations by O—H···O hydrogen bonds. The molecules are packed into infinite one dimensional chains along the [1 0 0] direction.

### Experimental

1-(2-hydroxyethyl)2-methyl-5-nitroimidazole) (0.350 g) [ALDRICH] was dissolved in 25 ml hot ethanol and silver nitrate [Sigma] (0.150 g) was dissolved in 20 ml ammonia solution in the molar ratio 2:1. These two solutions were mixed and heated under reflux for 48 h at a temperature of 363 K. Colourless plate shaped crystals were obtained after a month upon slow evaporation of the solvent.

### Refinement

After confirming their presence in the difference map, all H atoms were placed in calculated positions [C—H = 0.93 Å, CH_3_ = 0.96 Å CH_2_= 0.97Å and O—H = 0.86Å and refined using a riding model, with *U*_iso_(H) = 1.2*U*_eq_(C,*O*) and *U*_iso_(H) = -1.5*U*_eq_(methyl)]. The ratio of the occupancies for the major and minor components of the disordered nitro group O atoms were refined to 0.786 (3):0.214 (3). In the final refinement, this ratio was fixed at 0.8:0.2.

### Crystal data

**Table:**

[Ag(C_6_H_9_N_3_O_3_)_2_]NO_3_	*Z* = 2
*M**_r_* = 512.2	*F*_000_ = 516
Triclinic, *P*1	*D*_x_ = 1.882 Mg m^−^^3^
Hall symbol: -P 1	Mo *K*α radiation λ = 0.71073 Å
*a* = 6.6912 (1) Å	Cell parameters from 9959 reflections
*b* = 11.6846 (3) Å	θ = 3.2–37.8º
*c* = 12.9052 (3) Å	µ = 1.18 mm^−^^1^
α = 63.707 (1)º	*T* = 100.0 (1) K
β = 88.820 (1)º	Plate, colourless
γ = 87.486 (1)º	0.74 × 0.22 × 0.1 mm
*V* = 903.72 (3) Å^3^	

### Data collection

**Table:**

Bruker SMART APEXII CCD area-detector diffractometer	*R*_int_ = 0.026
φ and ω scans	θ_max_ = 32.5º
Absorption correction: multi-scan(SADABS; Bruker, 2005)	θ_min_ = 2.0º
*T*_min_ = 0.476, *T*_max_ = 0.892	*h* = −10→10
20384 measured reflections	*k* = −17→17
6509 independent reflections	*l* = −19→19
5958 reflections with *I* > 2σ(*I*)	

### Refinement

**Table:**

Refinement on *F*^2^	H-atom parameters constrained
Least-squares matrix: full	*w* = 1/[σ^2^(*F*_o_^2^) + (0.0281*P*)^2^ + 0.2414*P*] where *P* = (*F*_o_^2^ + 2*F*_c_^2^)/3
*R*[*F*^2^ > 2σ(*F*^2^)] = 0.024	(Δ/σ)_max_ = 0.005
*wR*(*F*^2^) = 0.059	Δρ_max_ = 0.58 e Å^−^^3^
*S* = 1.08	Δρ_min_ = −0.65 e Å^−^^3^
6509 reflections	Extinction correction: none
291 parameters	

### Special details

**Table:**

Geometry. Experimental. The low-temperature data was collected with the Oxford Crysosystem Cobra low-temperature attachement.All e.s.d.'s (except the e.s.d. in the dihedral angle between two l.s. planes) are estimated using the full covariance matrix. The cell e.s.d.'s are taken into account individually in the estimation of e.s.d.'s in distances, angles and torsion angles; correlations between e.s.d.'s in cell parameters are only used when they are defined by crystal symmetry. An approximate (isotropic) treatment of cell e.s.d.'s is used for estimating e.s.d.'s involving l.s. planes.

### Fractional atomic coordinates and isotropic or equivalent isotropic displacement parameters (Å^2^)

**Table:**

	*x*	*y*	*z*	*U*_iso_*/*U*_eq_	Occ. (<1)
Ag1	0.247504 (15)	0.147593 (9)	1.004773 (8)	0.01643 (3)	
O1	0.93316 (17)	0.44665 (10)	0.82229 (9)	0.0223 (2)	
H101	0.9052	0.3785	0.8594	0.027*	
O2	0.70475 (17)	0.41493 (11)	0.50145 (9)	0.0243 (2)	
O3	0.62322 (18)	0.21652 (11)	0.56769 (10)	0.0250 (2)	
O4	−0.46849 (17)	0.21717 (10)	1.24656 (9)	0.0214 (2)	
H1O4	−0.4288	0.2002	1.2003	0.026*	
O5	−0.18196 (16)	−0.16629 (10)	1.51008 (8)	0.0191 (2)	
O6	−0.10089 (17)	−0.26774 (9)	1.40801 (9)	0.0211 (2)	
N1	0.39023 (17)	0.26342 (10)	0.84333 (9)	0.01355 (19)	
N2	0.56794 (17)	0.41644 (10)	0.71061 (9)	0.01301 (19)	
N3	0.10790 (17)	0.07543 (11)	1.17193 (9)	0.0136 (2)	
N4	−0.07227 (16)	0.06598 (10)	1.32188 (9)	0.01194 (19)	
N5	0.63543 (18)	0.31651 (12)	0.57702 (10)	0.0168 (2)	
N6	−0.11678 (17)	−0.16962 (11)	1.42100 (9)	0.0139 (2)	
C1	0.02601 (19)	0.14301 (12)	1.22443 (11)	0.0127 (2)	
C2	−0.0527 (2)	−0.05563 (12)	1.32798 (11)	0.0124 (2)	
C3	0.0586 (2)	−0.04857 (12)	1.23573 (11)	0.0137 (2)	
H3A	0.0947	−0.1165	1.2192	0.016*	
C4	0.4512 (2)	0.22327 (12)	0.76340 (11)	0.0140 (2)	
H4A	0.4226	0.1455	0.7645	0.017*	
C5	0.5613 (2)	0.31599 (12)	0.68117 (11)	0.0135 (2)	
C6	0.46316 (19)	0.38035 (12)	0.80999 (11)	0.0133 (2)	
C7	0.4302 (2)	0.45784 (13)	0.87398 (13)	0.0190 (3)	
H7A	0.3141	0.4296	0.9223	0.028*	
H7B	0.4103	0.5461	0.8201	0.028*	
H7C	0.5449	0.4481	0.9211	0.028*	
C8	0.6977 (2)	0.52691 (12)	0.66141 (12)	0.0170 (2)	
H8A	0.6491	0.5913	0.685	0.02*	
H8B	0.6931	0.5642	0.5777	0.02*	
C9	0.9125 (2)	0.48636 (14)	0.70209 (13)	0.0188 (3)	
H9A	0.9564	0.4168	0.6844	0.023*	
H9B	0.9982	0.5574	0.6603	0.023*	
C10	−0.2072 (2)	0.10938 (13)	1.39020 (11)	0.0147 (2)	
H10A	−0.1702	0.1934	1.379	0.018*	
H10B	−0.1919	0.0513	1.4716	0.018*	
C11	−0.4241 (2)	0.11526 (13)	1.35576 (12)	0.0165 (2)	
H11A	−0.4541	0.0354	1.3545	0.02*	
H11B	−0.5093	0.1245	1.4135	0.02*	
C12	0.0442 (2)	0.28233 (13)	1.18237 (13)	0.0182 (3)	
H12A	0.0993	0.3183	1.1057	0.027*	
H12B	0.1306	0.2977	1.233	0.027*	
H12C	−0.0856	0.3213	1.1812	0.027*	
N7	0.25847 (17)	−0.14402 (10)	0.98555 (9)	0.0148 (2)	
O7A	0.2995 (2)	−0.22646 (13)	0.95060 (13)	0.0197 (3)	0.8
O8A	0.1492 (2)	−0.17829 (13)	1.07672 (11)	0.0195 (2)	0.8
O9A	0.3251 (2)	−0.03721 (13)	0.93965 (13)	0.0255 (3)	0.8
O7B	0.1786 (8)	−0.0844 (5)	1.0334 (4)	0.0190 (10)	0.2
O8B	0.3686 (7)	−0.0715 (5)	0.8893 (4)	0.0154 (9)	0.2
O9B	0.2298 (9)	−0.2500 (5)	0.9990 (5)	0.0163 (9)	0.2

### Atomic displacement parameters (Å^2^)

**Table:**

	*U*^11^	*U*^22^	*U*^33^	*U*^12^	*U*^13^	*U*^23^
Ag1	0.01378 (5)	0.01905 (5)	0.01297 (5)	−0.00266 (4)	0.00451 (3)	−0.00392 (4)
O1	0.0242 (5)	0.0180 (5)	0.0232 (5)	−0.0036 (4)	−0.0036 (4)	−0.0073 (4)
O2	0.0234 (5)	0.0286 (6)	0.0147 (4)	−0.0036 (4)	0.0057 (4)	−0.0041 (4)
O3	0.0304 (6)	0.0284 (6)	0.0222 (5)	−0.0004 (5)	0.0003 (4)	−0.0168 (4)
O4	0.0219 (5)	0.0204 (5)	0.0198 (5)	0.0055 (4)	−0.0027 (4)	−0.0075 (4)
O5	0.0193 (5)	0.0222 (5)	0.0135 (4)	−0.0019 (4)	0.0060 (4)	−0.0060 (4)
O6	0.0281 (6)	0.0133 (4)	0.0208 (5)	−0.0021 (4)	0.0040 (4)	−0.0065 (4)
N1	0.0130 (5)	0.0130 (5)	0.0132 (5)	−0.0023 (4)	0.0022 (4)	−0.0044 (4)
N2	0.0127 (5)	0.0115 (4)	0.0127 (4)	−0.0016 (4)	0.0002 (4)	−0.0033 (4)
N3	0.0131 (5)	0.0140 (5)	0.0124 (4)	−0.0002 (4)	0.0023 (4)	−0.0049 (4)
N4	0.0111 (5)	0.0135 (5)	0.0121 (4)	−0.0002 (4)	0.0009 (4)	−0.0065 (4)
N5	0.0141 (5)	0.0230 (6)	0.0128 (5)	0.0005 (4)	−0.0001 (4)	−0.0074 (4)
N6	0.0119 (5)	0.0149 (5)	0.0133 (5)	−0.0005 (4)	0.0008 (4)	−0.0048 (4)
C1	0.0107 (5)	0.0141 (5)	0.0134 (5)	−0.0006 (4)	0.0004 (4)	−0.0061 (4)
C2	0.0136 (5)	0.0114 (5)	0.0115 (5)	−0.0006 (4)	0.0013 (4)	−0.0044 (4)
C3	0.0148 (6)	0.0125 (5)	0.0126 (5)	0.0015 (4)	0.0013 (4)	−0.0046 (4)
C4	0.0146 (6)	0.0137 (5)	0.0137 (5)	−0.0022 (4)	0.0006 (4)	−0.0059 (4)
C5	0.0136 (5)	0.0151 (5)	0.0115 (5)	−0.0016 (4)	0.0008 (4)	−0.0056 (4)
C6	0.0119 (5)	0.0122 (5)	0.0142 (5)	−0.0002 (4)	0.0003 (4)	−0.0044 (4)
C7	0.0205 (6)	0.0159 (6)	0.0227 (6)	−0.0001 (5)	0.0030 (5)	−0.0106 (5)
C8	0.0173 (6)	0.0124 (5)	0.0172 (6)	−0.0055 (5)	0.0023 (5)	−0.0026 (4)
C9	0.0152 (6)	0.0185 (6)	0.0232 (6)	−0.0056 (5)	0.0028 (5)	−0.0094 (5)
C10	0.0154 (6)	0.0175 (6)	0.0135 (5)	0.0011 (5)	0.0022 (5)	−0.0091 (5)
C11	0.0138 (6)	0.0183 (6)	0.0167 (6)	0.0007 (5)	0.0030 (5)	−0.0072 (5)
C12	0.0188 (6)	0.0136 (5)	0.0221 (6)	−0.0023 (5)	0.0027 (5)	−0.0078 (5)
N7	0.0136 (5)	0.0157 (5)	0.0162 (5)	0.0004 (4)	−0.0016 (4)	−0.0082 (4)
O7A	0.0249 (7)	0.0191 (6)	0.0181 (6)	0.0024 (5)	−0.0005 (6)	−0.0111 (5)
O8A	0.0195 (6)	0.0248 (7)	0.0148 (5)	−0.0011 (5)	0.0033 (5)	−0.0095 (5)
O9A	0.0274 (7)	0.0148 (6)	0.0300 (7)	−0.0050 (5)	0.0031 (6)	−0.0059 (5)
O7B	0.018 (2)	0.026 (3)	0.020 (2)	0.003 (2)	0.0041 (19)	−0.017 (2)
O8B	0.013 (2)	0.016 (2)	0.013 (2)	−0.0043 (17)	0.0042 (17)	−0.0027 (17)
O9B	0.023 (3)	0.010 (2)	0.012 (2)	−0.0014 (19)	0.001 (2)	−0.0019 (17)

### Geometric parameters (Å, °)

**Table:**

Ag1—N3	2.1475 (11)	C4—C5	1.3646 (17)
Ag1—N1	2.1489 (11)	C4—H4A	0.93
O1—C9	1.4165 (18)	C6—C7	1.4791 (18)
O1—H101	0.7548	C7—H7A	0.96
O2—N5	1.2342 (15)	C7—H7B	0.96
O3—N5	1.2327 (16)	C7—H7C	0.96
O4—C11	1.4112 (17)	C8—C9	1.522 (2)
O4—H1O4	0.7478	C8—H8A	0.97
O5—N6	1.2372 (14)	C8—H8B	0.97
O6—N6	1.2299 (15)	C9—H9A	0.97
N1—C6	1.3487 (16)	C9—H9B	0.97
N1—C4	1.3583 (17)	C10—C11	1.5160 (19)
N2—C6	1.3505 (16)	C10—H10A	0.97
N2—C5	1.3871 (16)	C10—H10B	0.97
N2—C8	1.4738 (16)	C11—H11A	0.97
N3—C1	1.3430 (16)	C11—H11B	0.97
N3—C3	1.3638 (16)	C12—H12A	0.96
N4—C1	1.3561 (16)	C12—H12B	0.96
N4—C2	1.3884 (16)	C12—H12C	0.96
N4—C10	1.4742 (16)	N7—O9B	1.195 (5)
N5—C5	1.4206 (17)	N7—O7B	1.219 (5)
N6—C2	1.4182 (16)	N7—O9A	1.2207 (17)
C1—C12	1.4803 (18)	N7—O7A	1.2503 (17)
C2—C3	1.3643 (17)	N7—O8A	1.2854 (16)
C3—H3A	0.93	N7—O8B	1.374 (5)
			
N3—Ag1—N1	165.34 (4)	N2—C8—C9	110.63 (11)
C9—O1—H101	114.1	N2—C8—H8A	109.5
C11—O4—H1O4	109.2	C9—C8—H8A	109.5
C6—N1—C4	106.90 (10)	N2—C8—H8B	109.5
C6—N1—Ag1	127.13 (9)	C9—C8—H8B	109.5
C4—N1—Ag1	125.25 (9)	H8A—C8—H8B	108.1
C6—N2—C5	105.98 (10)	O1—C9—C8	112.19 (11)
C6—N2—C8	124.90 (11)	O1—C9—H9A	109.2
C5—N2—C8	127.79 (11)	C8—C9—H9A	109.2
C1—N3—C3	107.24 (10)	O1—C9—H9B	109.2
C1—N3—Ag1	127.60 (9)	C8—C9—H9B	109.2
C3—N3—Ag1	124.41 (9)	H9A—C9—H9B	107.9
C1—N4—C2	105.63 (10)	N4—C10—C11	111.72 (11)
C1—N4—C10	125.57 (11)	N4—C10—H10A	109.3
C2—N4—C10	127.52 (10)	C11—C10—H10A	109.3
O3—N5—O2	123.88 (12)	N4—C10—H10B	109.3
O3—N5—C5	116.63 (11)	C11—C10—H10B	109.3
O2—N5—C5	119.48 (12)	H10A—C10—H10B	107.9
O6—N6—O5	123.70 (11)	O4—C11—C10	112.83 (11)
O6—N6—C2	116.89 (11)	O4—C11—H11A	109
O5—N6—C2	119.40 (11)	C10—C11—H11A	109
N3—C1—N4	110.81 (11)	O4—C11—H11B	109
N3—C1—C12	124.38 (11)	C10—C11—H11B	109
N4—C1—C12	124.80 (11)	H11A—C11—H11B	107.8
C3—C2—N4	108.17 (11)	C1—C12—H12A	109.5
C3—C2—N6	125.67 (12)	C1—C12—H12B	109.5
N4—C2—N6	125.76 (11)	H12A—C12—H12B	109.5
N3—C3—C2	108.13 (11)	C1—C12—H12C	109.5
N3—C3—H3A	125.9	H12A—C12—H12C	109.5
C2—C3—H3A	125.9	H12B—C12—H12C	109.5
N1—C4—C5	108.62 (11)	O9B—N7—O7B	129.1 (4)
N1—C4—H4A	125.7	O9B—N7—O9A	157.0 (3)
C5—C4—H4A	125.7	O7B—N7—O9A	73.6 (3)
C4—C5—N2	107.74 (11)	O9B—N7—O7A	34.6 (3)
C4—C5—N5	126.13 (12)	O7B—N7—O7A	163.2 (3)
N2—C5—N5	125.74 (11)	O9A—N7—O7A	122.53 (14)
N1—C6—N2	110.75 (11)	O9B—N7—O8A	82.5 (3)
N1—C6—C7	124.08 (12)	O7B—N7—O8A	47.6 (3)
N2—C6—C7	125.16 (11)	O9A—N7—O8A	120.45 (13)
C6—C7—H7A	109.5	O7A—N7—O8A	116.95 (13)
C6—C7—H7B	109.5	O9B—N7—O8B	115.2 (4)
H7A—C7—H7B	109.5	O7B—N7—O8B	114.5 (4)
C6—C7—H7C	109.5	O9A—N7—O8B	41.9 (2)
H7A—C7—H7C	109.5	O7A—N7—O8B	80.9 (2)
H7B—C7—H7C	109.5	O8A—N7—O8B	162.1 (2)
			
N3—Ag1—N1—C6	8.8 (2)	Ag1—N1—C4—C5	−170.95 (9)
N3—Ag1—N1—C4	177.85 (15)	N1—C4—C5—N2	−0.29 (16)
N1—Ag1—N3—C1	17.4 (2)	N1—C4—C5—N5	−173.38 (13)
N1—Ag1—N3—C3	−173.76 (15)	C6—N2—C5—C4	0.52 (15)
C3—N3—C1—N4	1.10 (15)	C8—N2—C5—C4	167.74 (12)
Ag1—N3—C1—N4	171.44 (9)	C6—N2—C5—N5	173.64 (13)
C3—N3—C1—C12	−179.92 (13)	C8—N2—C5—N5	−19.1 (2)
Ag1—N3—C1—C12	−9.6 (2)	O3—N5—C5—C4	−10.8 (2)
C2—N4—C1—N3	−1.28 (15)	O2—N5—C5—C4	168.30 (14)
C10—N4—C1—N3	−169.09 (12)	O3—N5—C5—N2	177.36 (13)
C2—N4—C1—C12	179.75 (13)	O2—N5—C5—N2	−3.6 (2)
C10—N4—C1—C12	11.9 (2)	C4—N1—C6—N2	0.39 (15)
C1—N4—C2—C3	0.96 (15)	Ag1—N1—C6—N2	171.07 (9)
C10—N4—C2—C3	168.46 (12)	C4—N1—C6—C7	179.94 (13)
C1—N4—C2—N6	174.09 (13)	Ag1—N1—C6—C7	−9.39 (19)
C10—N4—C2—N6	−18.4 (2)	C5—N2—C6—N1	−0.57 (15)
O6—N6—C2—C3	−13.3 (2)	C8—N2—C6—N1	−168.26 (12)
O5—N6—C2—C3	165.71 (13)	C5—N2—C6—C7	179.89 (13)
O6—N6—C2—N4	174.73 (13)	C8—N2—C6—C7	12.2 (2)
O5—N6—C2—N4	−6.2 (2)	C6—N2—C8—C9	92.52 (15)
C1—N3—C3—C2	−0.46 (16)	C5—N2—C8—C9	−72.45 (17)
Ag1—N3—C3—C2	−171.18 (9)	N2—C8—C9—O1	−66.96 (14)
N4—C2—C3—N3	−0.32 (16)	C1—N4—C10—C11	94.80 (15)
N6—C2—C3—N3	−173.46 (12)	C2—N4—C10—C11	−70.35 (17)
C6—N1—C4—C5	−0.05 (15)	N4—C10—C11—O4	−71.90 (14)

### Hydrogen-bond geometry (Å, °)

**Table:**

*D*—H···*A*	*D*—H	H···*A*	*D*···*A*	*D*—H···*A*
O1—H101···O9B^i^	0.75	2.01	2.685 (5)	150
O1—H101···O8A^i^	0.75	2.15	2.8872 (17)	164
O4—H1O4···O7A^ii^	0.75	2.02	2.7225 (18)	158
O4—H1O4···O8B^ii^	0.75	2.29	2.985 (5)	156
C3—H3A···O8A	0.93	2.27	3.0820	145
C4—H4A···O9A	0.93	2.42	3.0269	122
C8—H8B···O2	0.97	2.35	2.891 (2)	115
C10—H10B···O5	0.97	2.36	2.888 (2)	113

Symmetry codes: (i) −*x*+1, −*y*, −*z*+2; (ii) −*x*, −*y*, −*z*+2.
